# Eosinophilic Colitis in an Adult Patient: A Case Report

**DOI:** 10.7759/cureus.90099

**Published:** 2025-08-14

**Authors:** Carter R Olberding, William Adili, Michael Herman

**Affiliations:** 1 Medicine, Lake Erie College of Osteopathic Medicine, Bradenton, USA; 2 Gastroenterology, Borland Groover, Jacksonville, USA

**Keywords:** abdominal pain, chronic diarrhea, colon biopsy, colonic inflammation, eosinophilic colitis, eosinophilic gastrointestinal disorders

## Abstract

Eosinophilic colitis (EC) is a rare inflammatory condition that often mimics irritable bowel syndrome (IBS) or inflammatory bowel disease (IBD), making it difficult to diagnose. It is characterized by significant eosinophilic infiltration in the colon, leading to symptoms like abdominal pain, diarrhea, and constipation. Herein, we present the case of a 43-year-old woman struggling with severe, episodic abdominal pain and unpredictable bowel habits, whose colonoscopy appeared grossly normal. Yet, mucosal biopsies revealed severe eosinophilic infiltration (>100 eosinophils per high-power field) in the cecum and ascending colon, with significant eosinophilia also noted in the transverse colon. These findings confirmed the diagnosis of eosinophilic colitis. This case underscores the rare but important presentation of EC with endoscopically normal mucosa and highlights how biopsy can be the only clue to an otherwise elusive diagnosis.

## Introduction

Many people with chronic digestive issues are initially told they have irritable bowel syndrome (IBS), especially when their symptoms do not fit neatly into a diagnosis like Crohn's disease or ulcerative colitis [1]. Eosinophilic colitis is one of those underrecognized conditions that can cause chronic, frustrating symptoms that affect a person’s daily life. Eosinophilic colitis is a rare condition, affecting approximately two to three individuals per 100,000 in the United States. However, it is often overlooked in clinical practice, particularly among patients evaluated for chronic diarrhea [2]. It is part of a group of disorders called eosinophilic gastrointestinal diseases (EGIDs), where an abnormal number of eosinophils (cells involved in allergic reactions and inflammation) accumulate in the digestive tract [3]. While eosinophilic esophagitis is widely recognized, eosinophilic colitis remains much less understood [4].

## Case presentation

Our patient is a 43-year-old woman who came to the clinic frustrated by a year-long struggle with severe abdominal pain and unpredictable bowel habits. The pain was mostly in her right upper quadrant but would sometimes radiate towards her left upper quadrant. The patient found it difficult to control the pain, but she admitted to receiving partial relief by often lying in the prone position to sleep. She described her bowel movements as erratic, alternating between constipation and diarrhea, often with an urgent need to defecate when she was out in public or during times of stress. She also noticed that consuming greasy foods was a trigger for her symptoms.

**Figure 1:**
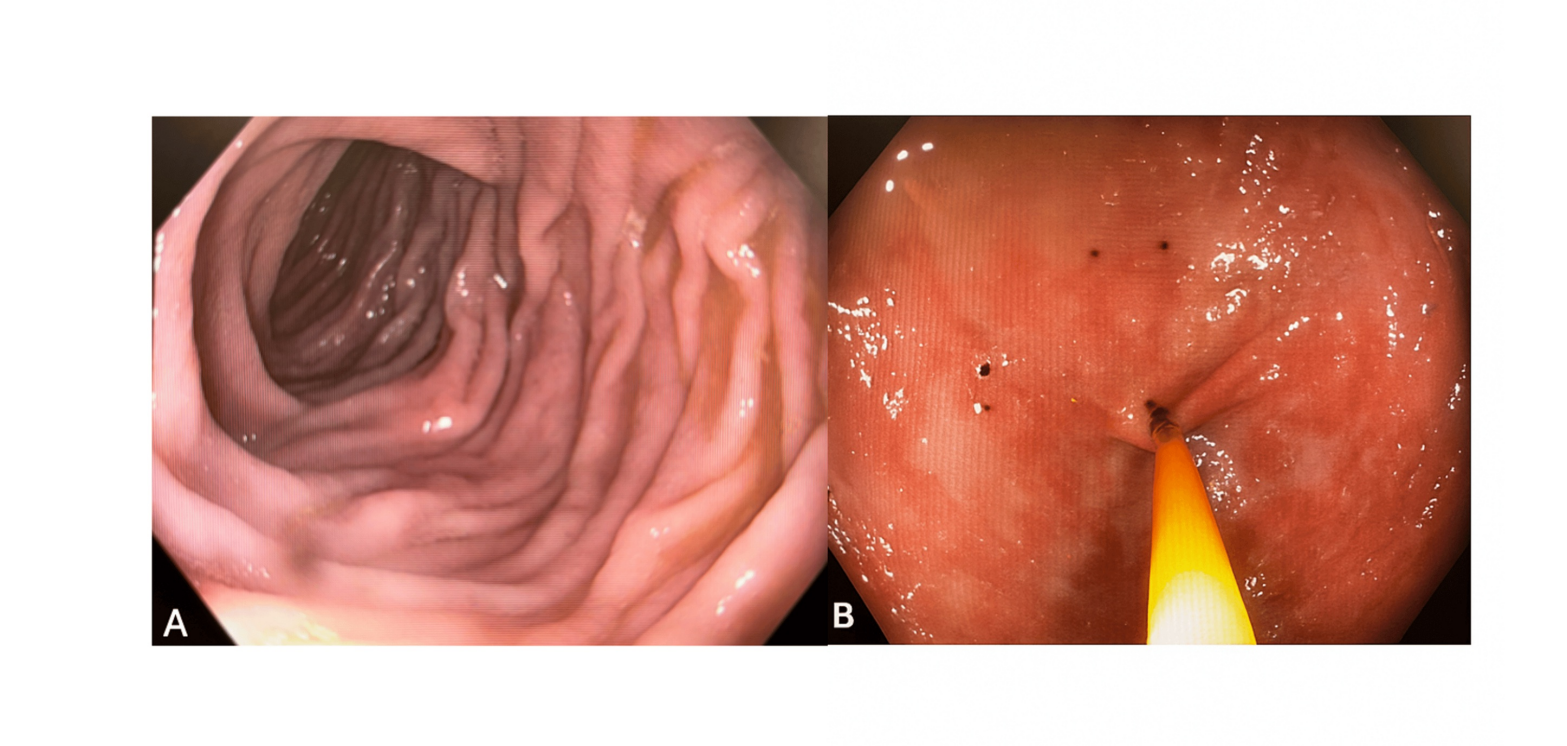
(A) An endoscopic image reveals normal colonic mucosa with a preserved vascular pattern and no evidence of inflammation, polyps, or mucosal abnormalities. (B) Colonoscopy reveals patchy areas in the colon with mucosal edema and punctate erythema around the biopsy site.

Another major issue for her was persistent acid reflux, leaving her with a strange taste in her mouth, and she received little relief with Famotidine (Pepcid). Concerned for an alternative diagnosis, she was referred for an upper endoscopy (EGD) and a colonoscopy with biopsies. Her EGD showed chronic gastritis but was negative for *Helicobacter pylori*. The colonoscopy results provided more answers, as multiple biopsies revealed severe eosinophilic infiltration (>100 eosinophils per high-power field) in the cecum and ascending colon, with crypt distortion. Additionally, prominent eosinophils (>75 eos/hpf) in the transverse colon were observed. Grossly, the patient's colonoscopy revealed minimal inflammation, erythema, or abnormal patterns. Figure 1 depicts the gross colonoscopic images.

At this point, treatment options were discussed with the patient, which included the mainstay of treatment, dietary modifications. The patient elected to pursue an elimination diet to pinpoint the source of food sensitivities. Other treatment options, including the possibility of corticosteroids followed by leukotriene receptor antagonists or antihistamines, were discussed with the patient if there was no response to dietary modification. Ultimately, at the time of publication, the patient was lost to follow-up and did not revisit the clinic.

## Discussion

Eosinophilic colitis can easily be mistaken for IBS or mild IBD (inflammatory bowel disease), but the hallmark finding, a dense accumulation of eosinophils in the colon, helps distinguish it [5]. It is important to note that eosinophilic colitis can affect patients at any age, but the majority of cases are reported between the third and fifth decades of life [6]. In this patient, her alternating bowel habits and food-related triggers could suggest an allergic or inflammatory component, which aligns with eosinophilic disease. Unlike Crohn's disease, eosinophilic colitis typically does not cause deep ulcers or strictures, but it can still cause significant pain, diarrhea, and malabsorption [5]. Many cases go undiagnosed because doctors do not routinely take biopsies in patients suspected of having IBS, with only an estimated 30% of gastroenterologists requesting eosinophil counts on tissue biopsies [7].

Histologically, eosinophilic colitis is characterized by a markedly increased eosinophil count in the colonic mucosa, often exceeding 20 eos/hpf, which distinguishes it from normal or IBS-affected tissue [3]. The clinical presentation can be highly variable, ranging from mild, intermittent symptoms to severe inflammation with protein-losing enteropathy [8]. Unlike Crohn's disease, which involves transmural inflammation leading to fibrosis and strictures, eosinophilic colitis primarily affects the mucosal layer, explaining the absence of deep ulcerations or fibrotic complications [9]. Furthermore, delayed diagnosis is common, in part due to the subtle endoscopic findings; the colon often appears normal or only mildly erythematous, underscoring the importance of routine biopsies in patients with chronic gastrointestinal symptoms. Early recognition and treatment, typically with corticosteroids or dietary modification in the form of elimination or elemental diets, can lead to significant symptomatic improvement and prevent complications associated with prolonged inflammation [5].

## Conclusions

Because of its nonspecific presentation and normal-looking colonoscopy, eosinophilic colitis is a commonly disregarded cause of persistent gastrointestinal symptoms that is frequently confused with functional disorders like IBS. In patients with persistent abdominal pain and changes in bowel habits, this case emphasizes the diagnostic value of mucosal biopsies, especially when conventional evaluations are not conclusive. Increased clinical awareness and early histologic confirmation are crucial for ensuring appropriate treatment, which can significantly enhance patient outcomes and alleviate troublesome symptoms in patients presenting with eosinophilic colitis.
